# Sleep disruption as a potential contributor to the worsening of eating disorder pathology during the COVID-19-pandemic

**DOI:** 10.1186/s40337-022-00704-9

**Published:** 2022-11-23

**Authors:** Jacqueline B. Mehr, Morgan H. James

**Affiliations:** 1 Department of Psychiatry, Robert Wood Johnson Medical School, Rutgers University and Rutgers Biomedical Health Sciences, Office 164, 683 Hoes Lane West, Piscataway, NJ 08854 USA; 2 Brain Health Institute, Rutgers University and Rutgers Biomedical and Health Sciences, Piscataway, NJ USA

**Keywords:** SARS-CoV-2, Binge eating disorder, Bulimia nervosa, Anorexia nervosa, Other specified feeding and eating disorders, Insomnia, Sleeplessness, Anxiety, Depression

## Abstract

The acute phase of the COVID-19 pandemic was associated with significant increases in the prevalence and severity of eating disorders (EDs). Studies also highlighted changes to sleep quality and duration in many individuals throughout this period. Although these two phenomena have been examined separately, here we highlight the need to investigate the potential link between these outcomes. Sleep dysregulation and EDs have previously been hypothesized to interact via a positive feedback loop, wherein poor sleep exacerbates ED symptomatology which, in turn, further worsens sleep. Thus, we speculate that the aggravation of sleep disturbances and EDs during COVID-19 lockdowns may have been somewhat interdependent. We further hypothesize that the worsening of depression and anxiety symptomology during the acute phase of the pandemic may have served as an additional mediating variable. Altogether, in our view, these observations highlight a need for future work to examine the possible causal relationship between sleep and ED pathology, which may ultimately lead to improved clinical management of disordered eating.

## Introduction

Research from the past two years has highlighted the significant negative impact of COVID-19 on the prevalence and severity of eating disorders (EDs) [1–4]. Pooled data indicate substantial increases in hospital admissions and ED severity relative to pre-pandemic timepoints, as well as increased anxiety and depression symptoms in ED patients. In qualitative studies, patients identify several potential contributing factors, including changes in routine, heightened isolation, increased exposure to triggering messages online, and treatment disruptions. Here, we highlight evidence that points to a potential association between pandemic-related disruptions to sleep patterns and the exacerbation of ED symptomology. Although partially encompassed by previously identified factors such as ‘changes in routine’ [1–3], we argue that sleep dysregulation represents a potential unique contributor that warrants closer examination in its own right. In this way, the pandemic may have shed light on a powerful variable affecting ED symptomology that has, until now, remained relatively underappreciated.

Pooled data across multiple studies indicate a striking ~ 50% increase in hospital admissions for EDs during the pandemic relative to prior timepoints [1]. More than 1 in 3 studies reported increased ED symptoms during this time, most notably increased frequency of binge eating episodes in binge eating disorder (BED) and bulimia nervosa (BN), as reported by both patients and their health care providers [1]. Concurrently, a separate literature indicates that the acute phase of the pandemic was associated with drastic reductions in sleep quality for many respondents [5, 6]. In one study that included over 20,000 people from 14 countries, approximately 1 in 5 individuals reported worsened sleep quality   throughout the pandemic, and these issues were  most pronounced in countries where the pandemic was escalating at the time of data collection [5]. Similarly, another international large-scale study (n = 22,330) reported rates of insomnia and insomnia disorder at 36.7% and 17.4% respectively, which are markedly higher compared to those reported in pre-pandemic studies [6]. Results from several other sleep studies sampling individuals on a national level across multiple age groups generally corroborate these findings [7–10]. Although very few studies examined ED and sleep outcomes in the same populations during the pandemic, one study found that 63.6% of former inpatients with bulimia nervosa reported sleep disturbances during the acute phase of the pandemic [11], indicating a potential link between these phenomena.

There is a known, but severely underexplored, association between poor sleep and eating outcomes. Disordered eating patterns are predictive of future sleep disturbances, and insomnia in adolescents is associated with an increased risk of EDs [12]. Dysregulated sleep is disproportionately observed in EDs characterized by excessive food intake (i.e. BED and BN), and is specifically linked to the dysregulated patterns of food intake that characterize these disorders rather than body weight per se, as these relationships are upheld when controlling for weight status (reviewed in [13]. Several factors are hypothesized to contribute to the relationship between sleep dysregulation and EDs. Eating pathology itself might influence sleep via gut microbiota-immune-brain interactions [14] or circadian entrainment of neuronal systems involved in reward and arousal [13, 15﻿]. Poor sleep has also been hypothesized to contribute to increased food craving and intake via dysregulation of neural networks involved in reward and executive control [13]. Similarly, interindividual variability of sleep timing and circadian phase might influence eating patterns [16]. Thus, the interrelationship between dysregulated sleep and eating is likely bidirectional/cyclical, and we posit that the exacerbation of these outcomes during the COVID-19 pandemic was, at least in part, interdependent. We suggest that this may have been especially apparent during lockdown periods, when negative outcomes associated with both ED and sleep were intensified [1, 6]. Curiously, some studies reported that the pandemic was associated with an improvement in sleep in a minority of participants [5], which parallels evidence that some patients reported an improvement in their ED symptomology during this time [1, 17, 18]. Although these improvements have been attributed to factors such as increased worktime flexibility and the emergence of efficacious online treatment options of EDs, it is possible that these improvements were in part also linked to improvements in sleep outcomes.

It is also notable that several studies indicate that depression and anxiety symptoms were exacerbated in ED patients over the course of the pandemic [1–4]. Depression and anxiety are bi-directionally linked to both ED and sleep outcomes [12], and anxiety and stress have been posited as potential causes or risk factors of worsened sleep in the COVID-19 pandemic [5, 7]. Thus, in addition to directly exacerbating ED outcomes via mechanisms mentioned above, we suggest that sleep disturbances may have *indirectly* contributed to ED trends by worsening depression and anxiety outcomes. Together, we believe that these observational data should inform more targeted studies that begin to test for causality (and directionality) between these factors. These studies might include monitoring sleep in human patients during ED inpatient treatments, actigraphy measures during outpatient treatment, and testing the efficacy of combining ED and depression/anxiety treatments with sleep-based therapies. Retrospective studies specifically designed to examine sleep, depression/anxiety, and ED outcomes in the same populations may also be helpful. These studies might also guide studies in laboratory animals to identify the neurobiological mechanisms linking sleep and ED symptoms, which has important implications for the development of medications to better manage these conditions. We note however, that animal models do not fully recapitulate the complex social and cognitive components of EDs nor depression/anxiety, and thus must be interpreted with these limitations in mind [19].

Finally, if sleep, depression/anxiety, and ED outcomes are truly interrelated, then it might be expected that these indices will improve concurrently as COVID-19 transitions to an endemic stage and there is a return to a ‘new normal’. Indeed, evidence collected to date indicates that changes in sleep and ED severity were most drastic at timepoints when the pandemic was worsening, with some improvements observed as the pandemic stabilized [5, 20]. Ongoing monitoring of sleep and ED outcomes during the ‘post-pandemic’ period will thus be informative for further exploring the interrelationship between these factors. Ideally, both sleep and ED indices can be measured in the same populations as a first step towards testing for potential causality between these factors. This monitoring may also be important for understanding whether the neurological sequalae of ‘long-Covid’ (21, 22) is associated with any persistent changes in ED symptomology.

## Conclusion

The pandemic has yielded an abundance of correlative data that collectively points to an association between drastic changes to daily routines and the worsening of ED symptomology. Although this phenomenon undoubtedly reflects a complex interaction of multiple contributing factors, we argue here that sleep dysregulation represents a potential common link. We note that this hypothesis is, at this point, highly speculative, as studies examining the causal link between sleep and EDs are extremely limited. To this end, we believe that these data highlight the need for a greater understanding of the mechanisms (neural or otherwise) through which dysregulated sleep might contribute to ED outcomes, and vice versa. Altogether, we suggest that efforts to better explore these phenomena might lead to better outcomes for ED patients via improved clinical management of sleep in the ‘post-pandemic world’.

## Data Availability

Not applicable.

## Abbreviations

ED: Eating disorder
BED: Binge eating disorder
BN: Bulimia nervosa
